# NAD^+^ metabolism as a target for metabolic health: have we found the silver bullet?

**DOI:** 10.1007/s00125-019-4831-3

**Published:** 2019-02-16

**Authors:** Niels J. Connell, Riekelt H. Houtkooper, Patrick Schrauwen

**Affiliations:** 1 Department of Nutrition and Movement Sciences, NUTRIM School for Nutrition and Translational Research in Metabolism, Maastricht University, Universiteitssingel 50, P.O. Box 616, 6200 MD Maastricht, the Netherlands; 2 Laboratory Genetic Metabolic Diseases, Amsterdam Gastroenterology and Metabolism, Amsterdam UMC, University of Amsterdam, Amsterdam, the Netherlands

**Keywords:** Diabetes, Energy metabolism, Human, Metabolic disease, NAD^+^, Review

## Abstract

NAD^+^ has gone in and out of fashion within the scientific community a number of times since its discovery in the early 1900s. Over the last decade, NAD^+^ has emerged as a potential target for combatting metabolic disturbances and the mitochondrial dysfunction that is mediated through sirtuin (SIRT) enzymes. The beneficial metabolic effects of the NAD^+^/SIRT axis have triggered an increased interest in NAD^+^ as an enhancer of energy metabolism. As a result, a myriad of publications have focused on NAD^+^ metabolism, with the majority of the work having been performed using in vitro models, and in vivo work largely consisting of interventions in *Caenorhabditis elegans* and rodents. Human intervention trials, on the other hand, are scarce. The aim of this review is to provide an overview of the state-of-the-art on influencing NAD^+^ metabolism in humans and to set the stage for what the future of this exciting field may hold.

**Electronic supplementary material:**

The online version of this article (10.1007/s00125-019-4831-3) contains a slideset of the figures for download, which is available to authorised users.

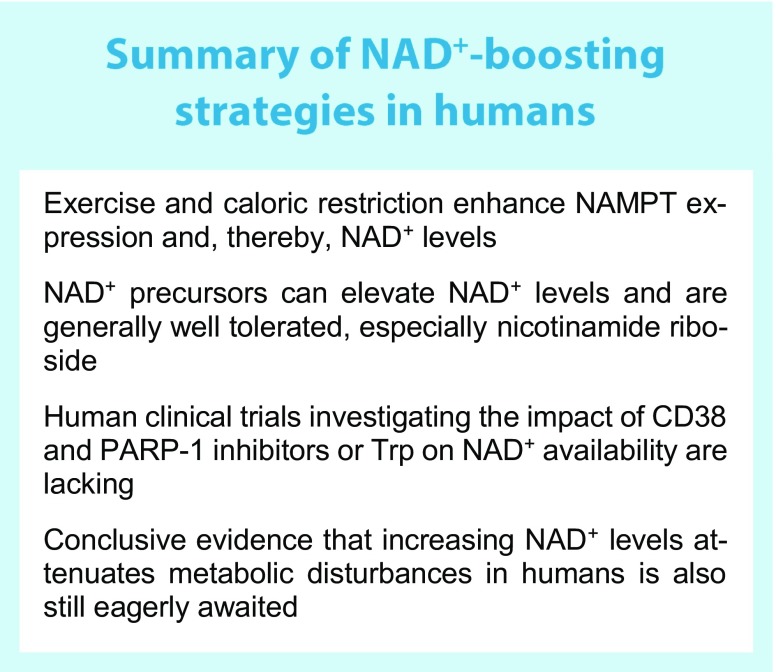

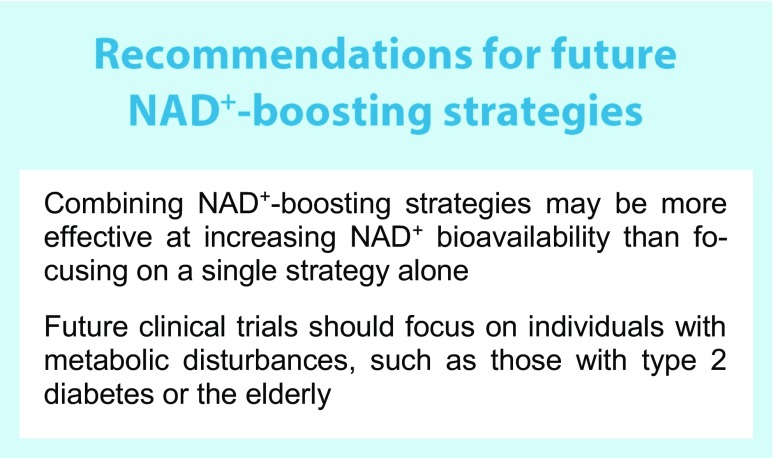





## Introduction

In recent years, a tremendous effort has been made to identify approaches for combatting metabolic disturbances and mitochondrial dysfunction, such as those seen in ageing [1] and type 2 diabetes mellitus [2, 3] by specifically targeting the sirtuin (SIRT) enzyme family [4]. SIRTs are NAD^+^-dependent deacetylating enzymes that regulate cellular metabolism [5]. To date, seven mammalian SIRT enzymes (SIRT1–7) have been identified, each having its own characteristic tissue and subcellular compartment expression, enzyme activity and targets. We kindly refer readers to Houtkooper et al [6] for a comprehensive review on SIRTs.

Several SIRT-targeting strategies have been deployed, demonstrating the metabolic benefits of SIRT activation. In mice, a SIRT1 gain-of-function mutation evoked a metabolic profile that protected against insulin-resistant diabetes by increasing hepatic insulin sensitivity, hepatic glucose tolerance and overall metabolic efficiency [7, 8]. Moreover, a proposed SIRT1 activator, SRT1720, increased mitochondrial respiration and improved insulin sensitivity [9], mimicking the signalling profile observed with caloric restriction [10] in high-fat-diet (HFD)-challenged mice. Resveratrol, an AMP-activated protein kinase (AMPK)-activating polyphenol that activates SIRT1, improved skeletal muscle mitochondrial function in healthy obese men, in individuals with type 2 diabetes and in first-degree relatives of those with type 2 diabetes, although the observed metabolic health effects are inconsistent [11, 12]. Together, these studies indicate that SIRT activation promotes metabolic health.

### Why NAD^+^?

The concept of influencing NAD^+^ bioavailability to activate the SIRTs was recently proposed for combatting metabolic disturbances and mitochondrial dysfunction in humans [13, 14]. This is supported by reports that decreased NAD^+^ bioavailability contributes to metabolic disturbances in ageing mice [15, 16] and humans [17, 18], and also in a rodent model of type 2 diabetes mellitus [16]. SIRTs are important consumers of NAD^+^ and depend on this rate-limiting substrate to act as metabolic sensors, responding to the level of available NAD^+^.

**Fig. 1:**
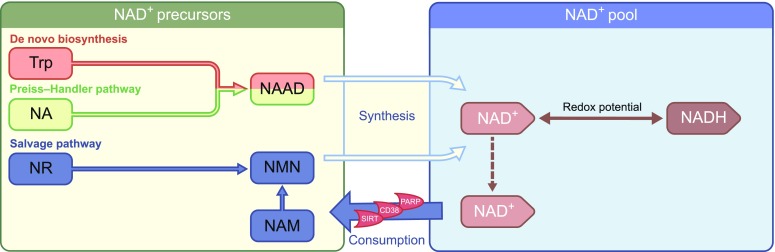
Summary of NAD^+^ metabolism. NAD^+^ can be synthesised from Trp through the de novo biosynthesis pathway in the liver and kidneys. Nicotinic acid (more commonly known as vitamin B_3_) enters the NAD^+^ pool through the Preiss–Handler pathway, whereas nicotinamide, nicotinamide riboside and NMN (re-)enter the NAD^+^ pool through the salvage pathway. NAD^+^ is consumed by SIRTs, CD38, and PARP enzymes, producing nicotinamide, which enters the pool of NAD^+^ precursors for resynthesis into NAD^+^. Dashed arrow, movement of NAD^+^ within the NAD^+^ pool. NA, nicotinic acid; NAAD, nicotinic acid adenine dinucleotide; NAM, nicotinamide; NR, nicotinamide riboside. This figure is available as part of a downloadable slideset

Considering the limited scope of this review, we will not digress into detail of the NAD^+^ metabolism and refer the reader to more comprehensive reviews on this topic [5, 19–21]. Briefly, however, as NADH is the predominant electron donor to the electron transport chain, NADH/NAD^+^ redox potential is an important indicator of the bioenergetic status of the cell and is tightly regulated [21]. The cytosolic and mitochondrial NADH/NAD^+^ and NADPH/NADP^+^ redox states are strongly connected. These states depend on the formation of NAD^+^ from NADH through cellular processes, such as the glycolytic enzyme activity, the citric acid cycle and the electron transport chain [20], thereby exemplifying the essentiality of NAD^+^ and its redox potential within cellular metabolism. The NAD^+^ pool is maintained through a continuous process of biosynthesis and breakdown, stemming from the salvage and the Preiss–Handler pathways or from de novo biosynthesis at one end, and enzymatic consumption at the other [20] (Fig. 1). When NAD^+^ levels rise, SIRTs activate and deac(et)ylate or mono-ADP-ribosylate a variety of metabolic substrates, such as peroxisome proliferator-activated receptor gamma coactivator 1α (PGC-1α) and forkhead box protein O1 (FOXO1). This elicits an array of metabolic adaptations, including mitochondrial biogenesis in skeletal muscle [19] and enhanced oxidative metabolism in skeletal muscle, brown adipose tissue and the liver [22, 23]. On a physiological level, this may lead to improved insulin sensitivity [24, 25], improved metabolic flexibility [26] and increased mitochondrial function [26, 27].

## NAD^+^ boosting strategies: preclinical evidence

### Exercise and caloric restriction induce nicotinamide phosphoribosyltransferase expression through AMPK

**Fig. 2:**
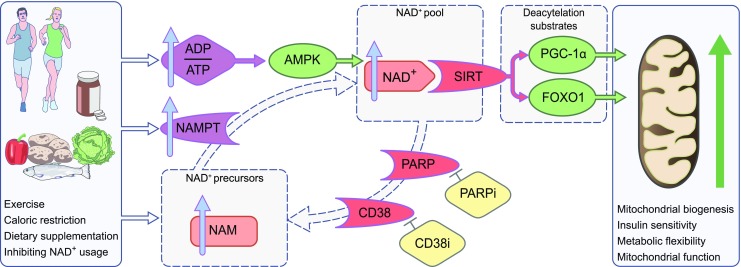
Effect of activating the NAD^+^/SIRT axis by increasing NAD^+^ bioavailability. Several approaches may be used to increase NAD^+^ bioavailability, including exercise, caloric restriction, dietary supplementation and inhibition of NAD^+^ consumption. These changes positively affect SIRT activation and subsequent PGC-1α and FOXO1 expression, resulting in mitochondrial changes and, as a consequence, metabolic adaptations. CD38i, CD38 inhibitor; FOXO1, forkhead box protein O1; NAM, nicotinamide; PARPi, PARP inhibitor. This figure is available as part of a downloadable slideset

Exercise and caloric restriction share a common denominator in that they affect AMPK activity, which can modulate NAD^+^ bioavailability (Fig. 2). To support this, AMPK activation in C2C12 myotubes increases cellular NAD^+^ levels and, in turn, activates SIRT1 and the subsequent PGC-1α-dependent upregulation of mitochondrial and lipid metabolism [28]. An increased demand for energy by the cell, such as during exercise, activates AMPK. With this in mind, it was shown that exercise induces the expression of nicotinamide phosphoribosyltransferase (NAMPT), the rate-limiting enzyme that converts nicotinamide into NAD^+^ [29], thereby increasing NAD^+^ bioavailability [30, 31]. The induction of NAMPT expression through AMPK has been suggested to be a mechanistic adaptation to the metabolic stress derived from both exercise and caloric restriction [32–34]. Moreover, exercise in rats has been demonstrated to induce de novo biosynthesis of NAD^+^ from l-tryptophan (Trp), ultimately increasing NAD^+^ bioavailability [35].

### NAD^+^ precursors increase NAD^+^ bioavailability and activate SIRTs

Various research groups have pursued sustained SIRT activation through an increase in endogenous NAD^+^ bioavailability. Preclinical research in ageing or HFD-challenged mice has shown that boosting NAD^+^ levels by supplementation with NAD^+^ precursors, such as nicotinamide mononucleotide (NMN) or nicotinamide riboside, attenuates age-related decline of muscle strength [1, 36], increases lifespan and healthspan [36]. In addition, oxidative metabolism and activation of SIRT1 and SIRT3 are enhanced in HFD-fed mice supplemented with NAD^+^ precursors [26]. In aged mice, NAD^+^ precursor supplementation also restored arterial SIRT1 activity, which was associated with improved vascular function and decreased aortic stiffness [37]. These findings demonstrate the feasibility of altering NAD^+^ bioavailability and subsequent SIRT activation.

More specifically, in HFD-fed mice, exogenous administration of the NAD^+^ precursor NMN was demonstrated to be a viable method of increasing endogenous NAD^+^ bioavailability and inducing SIRT1 activity, thereby attenuating the effects of the HFD and improving glucose tolerance and hepatic insulin sensitivity [16]. Long-term administration of NMN was also found to mitigate the age-associated decline in energy metabolism, insulin sensitivity and lipid metabolism [36]. Similarly, supplementation of HFD-challenged mice with nicotinamide riboside (another NAD^+^ precursor), also improved hepatic insulin sensitivity [26]. Additionally, an improved glucose tolerance and lipid profile were observed in mouse models of age-induced type 2 diabetes upon NMN supplementation [16].

The NAD^+^ precursors nicotinic acid and nicotinamide have also been used to supplement HFD-challenged mice, increasing hepatic NAD^+^ levels and improving glucose tolerance. In one study, nicotinamide proved to be a more potent booster of NAD^+^ than nicotinic acid as it was also found to specifically alter the expression of SIRT1, SIRT2 and SIRT6 [38]. Lastly, Acipimox, a synthetic nicotinic acid analogue, has been shown to elevate NAD^+^ in C2C12 myotubes [39].

Together, these preclinical data suggest that dietary supplementation of NAD^+^ precursors can increase NAD^+^ levels and beneficially affect metabolic health.

### Inhibition of NADases increases NAD^+^ bioavailability and SIRT1 activity

Preclinical research has explored compounds that can inhibit the NADases CD38 [40] and poly(ADP-ribose) polymerase-1 (PARP-1), reducing the enzymatic competition for their shared substrate, for example by reducing their NAD^+^-binding capacity, and thus enhancing SIRT1 activity (Fig. 2). Following this line of thought, a decrease in PARP-1 activity coincides with a rise in SIRT activity and NAD^+^ levels in worms [41] and mice [27], with *PARP-1*^−/−^ mice displaying a leaner phenotype with higher energy expenditure compared with *PARP-1*^+/+^ mice. In line with this, in skeletal muscle, PARP-1 inhibitor-induced increases in SIRT1 activity were accompanied by improved mitochondrial function, enhanced energy expenditure and endurance performance [42]. In endothelial progenitor cells, PARP-1 inhibition also preserved cellular NAD^+^ content [43]. Similarly, *Cd38* knockout mice have elevated NAD^+^ levels and are protected against HFD-induced metabolic inflexibility [44]. Moreover, the compounds apigenin, quercetin [45] and 78c [46] have all been demonstrated to enhance NAD^+^ levels and SIRT1 activity by inhibiting CD38.

## How to boost NAD^+^ in humans?

### Increasing NAD^+^ bioavailability through exercise and caloric restriction

Regular exercise and caloric restriction are well known to improve metabolic health in humans [47]. Alongside improving insulin sensitivity, metabolic flexibility and mitochondrial function, exercise also upregulates the expression of NAMPT in human skeletal muscle [48] (Fig. 2). Endurance-trained athletes have a twofold higher expression of NAMPT in skeletal muscle compared with baseline levels in sedentary obese, non-obese and type 2 diabetic individuals. After completing a 3 week training intervention, the non-obese group displayed increased NAMPT expression over baseline. NAMPT levels correlated positively with PGC-1α expression, mitochondrial content, maximal mitochondrial ATP synthesis in skeletal muscle and overall maximal aerobic capacity [48]. Concordantly, increased skeletal muscle SIRT3 content and PGC-1α expression were reported in men who were sedentary obese at baseline after a 12 week aerobic exercise intervention [49]. In a 6 week one-leg endurance exercise intervention, NAMPT protein levels only increased in the trained leg as compared with the untrained leg [34], further supporting the paradigm of activating the NAD^+^/SIRT axis through exercise and NAMPT induction.

Continuing, during a caloric restriction-induced weight-loss intervention, NAMPT and subsequent SIRT1 expression were found to be increased in adipose tissue of healthy obese participants [50] when compared with healthy lean participants. The participants were studied prior to, and after 5 months and 12 months, of the intervention, with the intervention resulting in a loss of 17.1% of body weight in the obese group. At baseline, gene expression of *SIRT1*, *SIRT3*, *SIRT7* and *NAMPT* were significantly lower and PARP-1 activity significantly higher in the obese participants when compared with the lean group, indicating a state of low NAD^+^ bioavailability in obese individuals. With weight loss, *SIRT1* expression increased, whereas PARP-1 activity declined in the subcutaneous adipose tissue of the obese group [50]. Evidence that a state of obesity or overnutrition indeed lowers NAD^+^ levels also comes from studies of longer-term overfeeding using an HFD for 8 weeks in young, healthy men. This resulted in reduced NAD^+^ levels and SIRT activity in skeletal muscle when compared with baseline [51]. This was further supported by PGC-1α hyperacetylation in the same skeletal muscle biopsies. Concurring with these findings, a study in young adult monozygotic twins (*n* = 26 obesity-discordant pairs and *n* = 14 obesity-concordant pairs) reported that obesity was associated with lower NAD^+^/SIRT axis activation in subcutaneous adipose tissue [14]. Together, these findings suggest that a state of energy abundance is prone to reduce the activity of the NAD^+^/SIRT axis and that inducing a state of energy demand may aid to restore NAD^+^ levels.

### Supplementation of NAD^+^ precursors

From a human dietary perspective, Trp, nicotinic acid, nicotinamide, and nicotinamide riboside are the predominant NAD^+^ precursors currently used in intervention trials, with nicotinamide riboside being the latest addition to the array of dietary NAD^+^ precursors (Fig. 1). The efficacy and safety of treatment with each of these NAD^+^ precursors are discussed in more detail below.

#### Nicotinamide

Phase 0 and phase 1 trials have demonstrated tolerance and safety of nicotinamide in daily pharmacological doses up to 3.5 g [52–56] and single doses of up to 6 g [57–59]. However, at doses above this, nicotinamide can become hepatotoxic [60].

#### Nicotinic acid and Acipimox

Nicotinic acid is the most effective pharmacological drug available for elevating HDL-cholesterol and lowering total cholesterol, LDL-cholesterol and triacylglycerol levels, thereby reducing the overall cardiovascular risk profile of the user [61]. However, nicotinic acid can elevate plasma glucose levels by inducing insulin resistance following a rebound increase in circulating NEFAs [62]. This poses a challenge when using nicotinic acid as (add-on to statin) therapy for dyslipidaemia in individuals with impaired glucose tolerance, impaired fasted glucose or type 2 diabetes, with the reduction in overall cardiovascular disease risk on one hand and compromised glycaemic control on the other. The worsening of hyperglycaemia with nicotinic acid use would possibly require additional therapeutic fine tuning to be implemented on an individual level to maintain glycaemic control. Alternatively, a reduction in the dose of nicotinic acid could improve glycaemic control, however, this may require acceptance of reciprocal compromise of the lipid profile or additional therapy to be initiated.

A large clinical trial evaluated the efficacy of nicotinic acid as a treatment for hypercholesterolaemia, with a daily dose of 1–3 g, for a duration of 96 weeks [63]. Overall, nicotinic acid was well tolerated. However, flushing was reported as a major adverse event. In contrast to nicotinamide, nicotinic acid is a vasoactive compound [64] and activates the G protein-coupled receptor, GPR109A, thereby inducing flushing [65]. In an attempt to reduce the occurrence of flushing and improve adherence, synthetic and extended- and sustained-release formulations of nicotinic acid were developed. Acipimox is a synthetic nicotinic acid analogue and, thereby, an NAD^+^ precursor that can be utilised by the Preiss–Handler pathway (Fig. 1). Although Acipimox displays the vasoactive properties that lead to flushing, we previously showed that treating individuals with type 2 diabetes with Acipimox for 2 weeks resulted in an improvement in skeletal muscle mitochondrial function [39]. In two other trials, Acipimox therapy improved insulin sensitivity [66, 67]. However, Acipimox is mainly used for lowering circulating NEFA levels and these human experiments do not allow us to conclude whether the beneficial effects observed were due to NAD^+^ boosting actions alone, although, in the first trial [39], the improved mitochondrial function with Acipimox therapy was accompanied with elevated (as opposed to lower) NEFA levels due to a known rebound effect. Unfortunately, the newer formulations of nicotinic acid have been associated with a higher occurrence of gastro-intestinal complaints, hepatotoxicity and hyperglycaemia, and a decreased HDL-cholesterol-raising efficacy compared with regular nicotinic acid [61]. Together, the side effects limit the use of nicotinic acid for further clinical exploration and implementation.

#### NADH

NADH supplementation has also been used to boost NAD^+^ levels in humans. In a small study, 80 adults with chronic fatigue syndrome received daily doses of 20 mg of NADH combined with 200 mg of coenzyme Q_10_ and were compared with placebo-treated individuals [68, 69]. The intervention improved reported fatigue [68] and increased maximal heart rate after 8 weeks of treatment [69] but did not alter body weight or blood pressure. Additionally, in peripheral blood mononuclear cells (PBMCs), the intervention significantly reduced NAD^+^ levels and increased NADH levels, thus, significantly lowering the NAD^+^/NADH ratio over baseline. Furthermore, ATP content and citrate synthase activity were significantly increased in PBMCs [68]. Unfortunately, it cannot be distinguished whether the observed results were solely attributed to NADH supplementation considering the co-administration of coenzyme Q_10_ in this study.

#### Nicotinamide riboside

In contrast to nicotinic acid, nicotinamide riboside is not vasoactive and does not cause flushing [70], thereby overcoming one of the adverse effects of nicotinic acid supplementation. In a recently published placebo-controlled, double-blind, randomised, phase 1 crossover trial, a daily dose of 1000 mg of nicotinamide riboside for 6 weeks was demonstrated to be well tolerated and adverse events were no more frequent than in the placebo arm [71]. These findings are confirmatory of the preceding phase 1 trials [72–74]. Additionally, nicotinic acid adenine dinucleotide (NAAD) has been confirmed as a reliable and sensitive biomarker for assessing changes in NAD^+^ levels following nicotinamide riboside supplementation [72].

Daily nicotinamide riboside supplementation of up to 2000 mg can effectively enhance blood NAD^+^ levels, achieving higher steady-state concentrations over baseline [73]. Concordantly, a more recent study demonstrated that nicotinamide riboside supplementation increased NAAD and NAD^+^ levels by ~60% in PBMCs. In this study, the effect of 6 weeks of nicotinamide riboside supplementation vs placebo was tested in healthy middle-aged and older adults. It was also found that 6 weeks of nicotinamide riboside supplementation tended to improve systolic blood pressure and pulse-wave velocity, both of which are markers of cardiovascular health [71]. However, no effect of nicotinamide riboside supplementation was found on physical performance outcomes, such as the 4 metre or 6 minute walk test, handgrip strength or maximum torque. Moreover, metabolic variables, such as $\dot{V}{\mathrm{O}}_{2\mathrm{max}}$ during a treadmill exhaustion test, respiratory exchange ratio, and insulin sensitivity assessed by an IVGTT, did not differ between the groups. From these findings, it was concluded that long-term nicotinamide riboside supplementation is a viable strategy for enhancing NAD^+^ in humans and potentially has cardiovascular benefits that require further exploration in larger trials.

Most recently, an RCT of daily treatment with 2000 mg of nicotinamide riboside for 12 weeks was reported, evaluating safety, insulin sensitivity and other metabolic variables in 40 healthy, obese, middle-aged men [75]. Overall, nicotinamide riboside was well tolerated and only four adverse events were reported: pruritus, excessive sweating, bloating and transient changes in stools. Nicotinamide riboside supplementation increased NAD^+^ metabolism, as was seen by an increase in urinary metabolites. Using the hyperinsulinaemic–euglycaemic clamp technique, insulin sensitivity was found to be unchanged before and after supplementation and when compared with the placebo condition. In addition, resting energy expenditure and respiratory exchange ratio were not affected by nicotinamide riboside supplementation. Also, intrahepatic lipid content and body composition remained unchanged in the treatment group vs baseline and compared with the placebo group. Finally, a significant but modest increase in serum triacylglycerol levels was detected after nicotinamide riboside supplementation when compared with baseline values. The authors concluded that this study was underpowered and future studies should be larger and focus on other variables of metabolic health, such as intrahepatic lipid content, which showed significant changes in rodents [76, 77] treated with nicotinamide riboside and approached significance in this study.

#### Tryptophan

Another dietary NAD^+^ precursor, Trp, is an essential amino acid and is metabolised into NAD^+^ through de novo biosynthesis in the liver and kidneys [20]. This route is critical for maintaining the NAD^+^ pool, even though the conversion ratio of Trp to NAD^+^ is low in humans, averaging 60:1 [78]. Nonetheless, Trp is deemed capable of meeting the metabolic demands of NAD^+^ metabolism in nicotinic acid- and nicotinamide-deficient diets, and is well tolerated at high doses, between 30 and 50 mg/kg bodyweight, apart from drowsiness/sleepiness [79].

Recently, higher circulating Trp levels were identified as a predictive marker for the development of type 2 diabetes in a large prospective Chinese cohort [80]. However, to date, no dietary supplementation studies are available that directly assess whether boosting NAD^+^ through Trp might be metabolically beneficial in humans.

### Inhibition of NAD^+^ consumers

The drawback of pharmacological strategies involving CD38 and PARP-1 inhibition is the original intended therapeutic use in malignancies [81, 82]. As such, no clinical trials with PARP-1 or CD38 inhibitors that focus on improving metabolic variables have been conducted in humans. This, however, does not imply that this strategy must be abandoned altogether, as a viable work-around to exploit the theoretical metabolic benefit of inhibition of NAD^+^ consumers may present itself in due time, allowing us to assess their efficacy in clinical trials.

## Future perspective

The current evidence base from preclinical research on NAD^+^ is setting the stage for trials in humans by identifying the points at which intervening in the NAD^+^ metabolism process seems to be clinically and physiologically relevant (see Summary text box). Even though many results have not been replicated in humans at this point in time, phase 0 and phase 1 trials have proven the feasibility and safety of NAD^+^ boosting in humans. As most evidence that increased NAD^+^ levels may be beneficial to human metabolism comes from indirect observations, such as exercise and weight loss interventions, the assessment of efficacy in well-powered phase 2 and phase 3 trials is urgently awaited in order to draw clear conclusions. Additionally, studies in metabolically disturbed individuals must be considered as these are more in line with the preclinical models used. To date, generally healthy populations have been included in studies in this area, in which the range of improvement may be too small to detect significant changes. The combination of strategies to increase NAD^+^, such as exercise, caloric restriction, or CD38 and PARP-1 inhibitors, with NAD^+^ precursor supplementation may also be considered, to evaluate added efficacy of such approaches, as seen in mice [15] (see Recommendations text box).

Currently, a number of clinical trials (Table 1) are underway in which NAD^+^ precursor supplementation is being used to improve (often disturbed) metabolic health variables. The coming years will prove whether the promising results observed in preclinical studies can indeed find human translation.

**Table 1:** Overview of clinical trials on NAD^+^ metabolism in humans

ClinicalTrials.gov registration no.	Title	Disease/condition	Interventions	Outcome measures	Sex	Participant age (years) and number	Study design
NCT03540758	Regulation of endogenous glucose production by central K_ATP_ channels	T2D; glucose metabolism disorders; high BG	Drugs: diazoxide ± nicotinic acid or placebo	EGP	M and F	21–65 (adult, older adult) *n* = 45	Randomised, SGA, single masked^a^, for basic science purposes
NCT03432871	Nicotinamide riboside and mitochondrial biogenesis	Mitochondrial diseases	Dietary supplement: nicotinamide riboside	Bioavailability; safety (treatment-related AEs, blood analytes, temperature, BP, pulse); mitochondrial biogenesis (MRI, respiratory chain enzyme analysis, mitochondrial DNA quantification); mitochondrial disease symptoms (dynamometric measure of muscle strength, 6 minute walk test, QOL [SF-36; qualitative], TUG)	M and F	18–70 (adult, older adult) *n* = 15	SGA, no masking (open label), for treatment purposes
NCT03310034	NAD supplementation study (NADS)	Ageing	Dietary supplement: NAD^+^ precursors (nicotinic acid, nicotinamide and Trp) or control	Ex vivo mitochondrial respiration; basal metabolic rate; in vivo mitochondrial capacity; submaximal exercise energy expenditure; glucose tolerance; ectopic lipid accumulation; acetylcarnitine levels; physical function	M and F	65–75 (older adult) *n* = 14	Randomised, crossover assignment, double masking^b^, for basic science purposes
NCT03151707	The effects of nicotinamide riboside supplementation on NAD^+^/NADH ratio and bioenergetics	Healthy	Drug: nicotinamide riboside	Brain NAD^+^/NADH ratio; brain PCr/ATP ratio; creatine kinase enzyme rate	M and F	18–65 (adult, older adult) *n* = 60	SGA, no masking (open label), for treatment purposes
NCT03151239	Effect of ‘nicotinamide mononucleotide’ (NMN) on cardiometabolic function	Glucose metabolism disorders	Dietary supplement: NMN or placebo	Insulin sensitivity; beta cell function	F	55–75 (adult, older adult) *n* = 50	Randomised, parallel assignment, triple masking^c^, for basic science purposes
NCT02950441	Nicotinamide adenine dinucleotide and skeletal muscle metabolic phenotype (NADMet)	Ageing	Dietary supplement: nicotinamide riboside or placebo	Mitochondrial function in skeletal muscle (high resolution respirometry); skeletal muscle NAD^+^ levels in vastus lateralis biopsy (targeted metabolomics); response to OGTT/HOMA-IR; lipid profile; muscle arterio-venous difference (tissue-specific metabolite trafficking, O_2_ consumption, CO_2_ production); muscle biopsy (adaptive expression profile [genomic]); RMR (indirect calorimetry); NAD^+^ metabolomics, changes in steroid ratios in 24 h urine collection (GC/MS); muscle strength (grip testing)	Male	70–80 (older adult) *n* = 12	Randomised, crossover assignment, quadruple masking^d^, for treatment purposes
NCT02835664^e^	Nicotinamide riboside and metabolic health	Obesity; insulin resistance	Dietary supplement: nicotinamide riboside or placebo	Muscle and liver insulin sensitivity; ex vivo muscle mitochondrial function; ectopic lipid accumulation; BAT activity; cardiovascular risk variables; whole body EE; body composition; acetylcarnitine levels	M and F	45–65 (adult, older adult) *n* = 15	Randomised, crossover assignment, quadruple masking^d^, for treatment purposes
NCT02689882^e^	Pharmacokinetic study of nicotinamide riboside	Metabolic disturbance	Dietary supplement: nicotinamide riboside	Average Css of nicotinamide riboside and NAD following up-titration to 1000 mg by mouth twice daily; serum levels of K^+^, creatine kinase, glucose, uric acid and ALT	M and F	21–50 (adult) *n* = 8	SGA, no masking (open label)
NCT02303483^e^	The effect of vitamin B_3_ on substrate metabolism, insulin sensitivity, and body composition in obese men	Obesity	Dietary supplement: nicotinamide riboside or placebo	Insulin sensitivity; substrate metabolism; body composition; activation of satellite cells; lipid accumulation in liver and skeletal muscle; glucose turnover; insulin signalling in skeletal muscle and adipose tissue biopsies; palmitate turnover; gut microbiota; incretin hormone secretion	M	40–70 (adult, older adult) *n* = 40	Randomised, parallel assignment, quadruple masking^d^, for treatment purposes
NCT02300740^e^	Pharmacokinetic analysis of nicotinamide riboside	Healthy	Dietary supplement: nicotinamide riboside	Serum nicotinamide riboside; metabolites of nicotinamide riboside; AUC for serum nicotinamide riboside; T_1/2_, C_max_ and T_max_ of serum nicotinamide riboside	M	18–30 (adult) *n* = 12	Randomised, crossover assignment, no masking (open label), for treatment purposes
NCT01321034^e^	Effect of niacin in the lipoprotein (a) concentration	Hypercholesterolaemia	Drug: niacin/laropiprant^f^	Absolute and relative Lp(a) lowering effect of niacin/laropiprant at 1 g/20 mg and 2 g/40 mg per day in participants with normal, high and very high Lp(a) (<1.07 μmol/l, 1.07–2.14 μmol/l and > 2.14 μmol/l, respectively) and depending on number of KIV-2 repeated copies on the apo(a) gene	M an F	18–80 (adult, older adult) *n* = 90	SGA, no masking (open label), for treatment purposes
NCT01216956^e^	Metabolic effects of an 8 week Niaspan treatment in patients with abdominal obesity and mixed dyslipidemia	Obesity and dyslipidaemia	Drug: ER nicotinic acid or placebo	NEFA and triacylglycerol concentrations over time; insulin sensitivity; lipoprotein metabolism; lipid profile	M	18–65 (adult, older adult) *n* = 24	Randomised, crossover assignment, double masking^b^, for treatment purposes
NCT00618995^e^	A study to evaluate the effects of ER niacin/laropiprant, laropiprant, ER niacin, and placebo on urinary prostanoid metabolites (0524A-079)	T2D	Drug: ER niacin + laropiprant^f^ or ER niacin or laropiprant^f^ or placebo	11-dTxB_2_; PGIM	M and F	18–65 (adult, older adult) *n* = 26	Randomised, crossover assignment, double masking^b^, for treatment purposes
NCT00485758^e^	Extended niacin/laropiprant in patients with type 2 diabetes (0524A-069)	T2D	Drug: ER niacin/laropiprant^f^ or placebo (unspecified)	LDL-c; HDL-c; triacyclglycerol	M and F	18–80 (adult, older adult) *n* = 796	Randomised, parallel assignment, double masking^b^, for treatment purposes

Data are from trials registered on ClinicalTrials.gov

^a^Participant only

^b^Participant and investigator

^c^Participant, care provider and investigator

^d^Participant, care provider, investigator and outcomes assessor

^e^Trial has been completed

^f^Laropiprant: prostaglandin D_2_ receptor subtype DP_1_ receptor antagonist that combats nicotinic acid-induced flushing

AE, adverse event; ALT, alanine aminotransferase; BAT, brown adipose tissue; BG, blood glucose; Css, steady-state concentration; 11-dTxB_2_, urinary 11-dehydrothromboxane B_2_; EE, energy expenditure; EGP, endogenous glucose production; ER, extended-release; F, female; HDL-c, HDL-cholesterol; KIV-2, kringle IV type 2; LDL-c, LDL-cholesterol; Lp(a), lipoprotein(a); M, male; MRI, magnetic resonance imaging; PCr, phosphocreatine; PGIM, prostaglandin I metabolite; QOL, quality of life; RMR, resting metabolic rate; SF-36, short form (36) health survey questionnaire; SGA, single group assignment; T2D, type 2 diabetes; TUG, timed up and go test

## Electronic supplementary material

ESM (PPTX 403 kb)

## Abbreviations

AMPK: AMP-activated protein kinase
HFD: High-fat diet
NAMPT: Nicotinamide phosphoribosyltransferase
NMN: Nicotinamide mononucleotide
PARP-1: Poly(ADP-ribose) polymerase-1
PBMC: Peripheral blood mononuclear cell
PGC-1α: Peroxisome proliferator-activated receptor gamma coactivator 1α
SIRT: Sirtuin
Trp: l-Tryptophan
