# Supplementary material for: NAD+ metabolism as a target for metabolic health: have we found the silver bullet?
Source: Diabetologia. 2019 Feb 16;62(6):888–99. doi: 10.1007/s00125-019-4831-3 (PMC6509089; doi:10.1007/s00125-019-4831-3)
Supplement: Supplementary file 1 — (PPTX 403 kb) [file 125_2019_4831_MOESM1_ESM.pptx]

## Slide 1
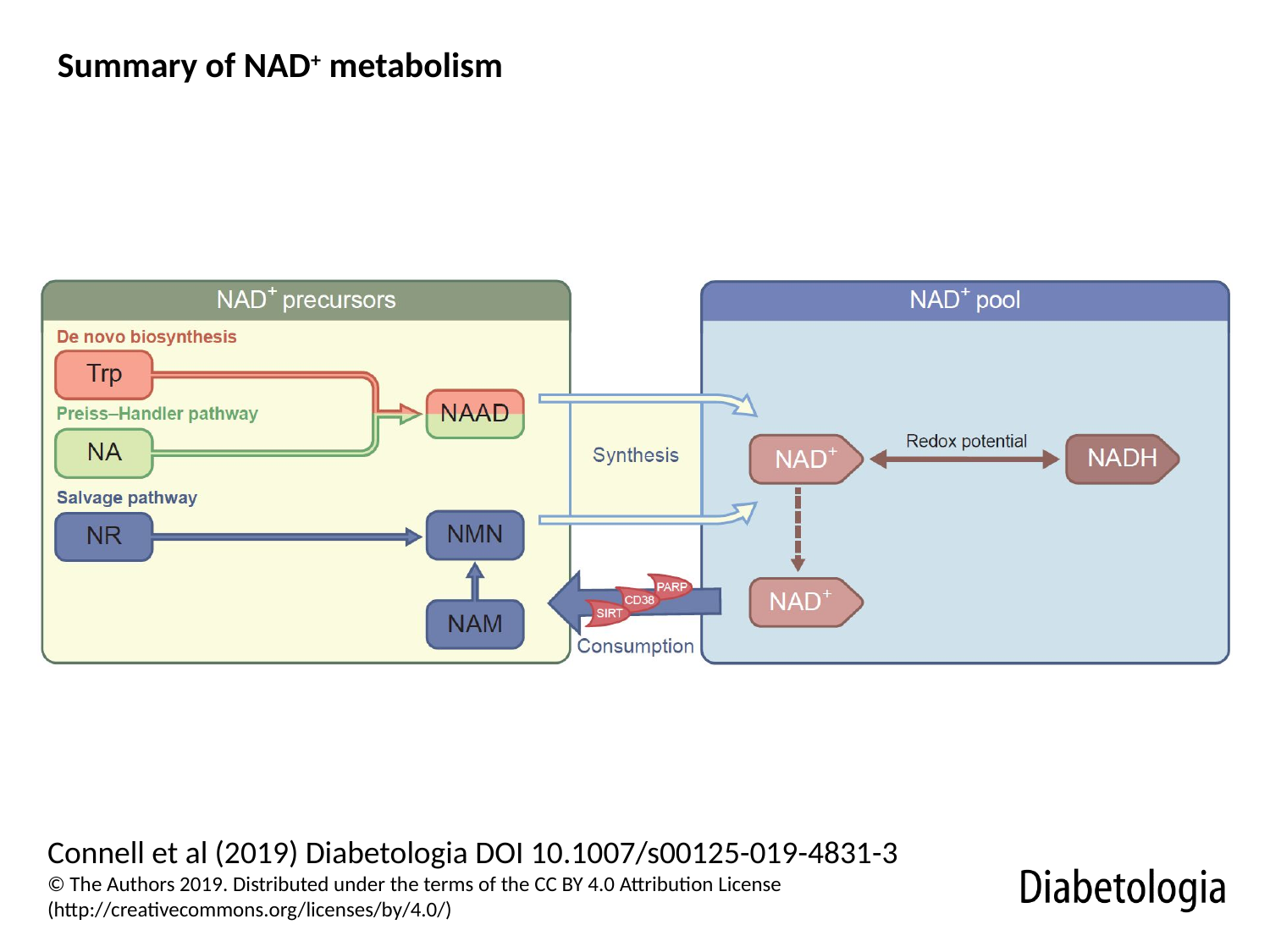

Summary of NAD+ metabolism
Connell et al (2019) Diabetologia DOI 10.1007/s00125-019-4831-3
© The Authors 2019. Distributed under the terms of the CC BY 4.0 Attribution License (http://creativecommons.org/licenses/by/4.0/)

## Slide 2
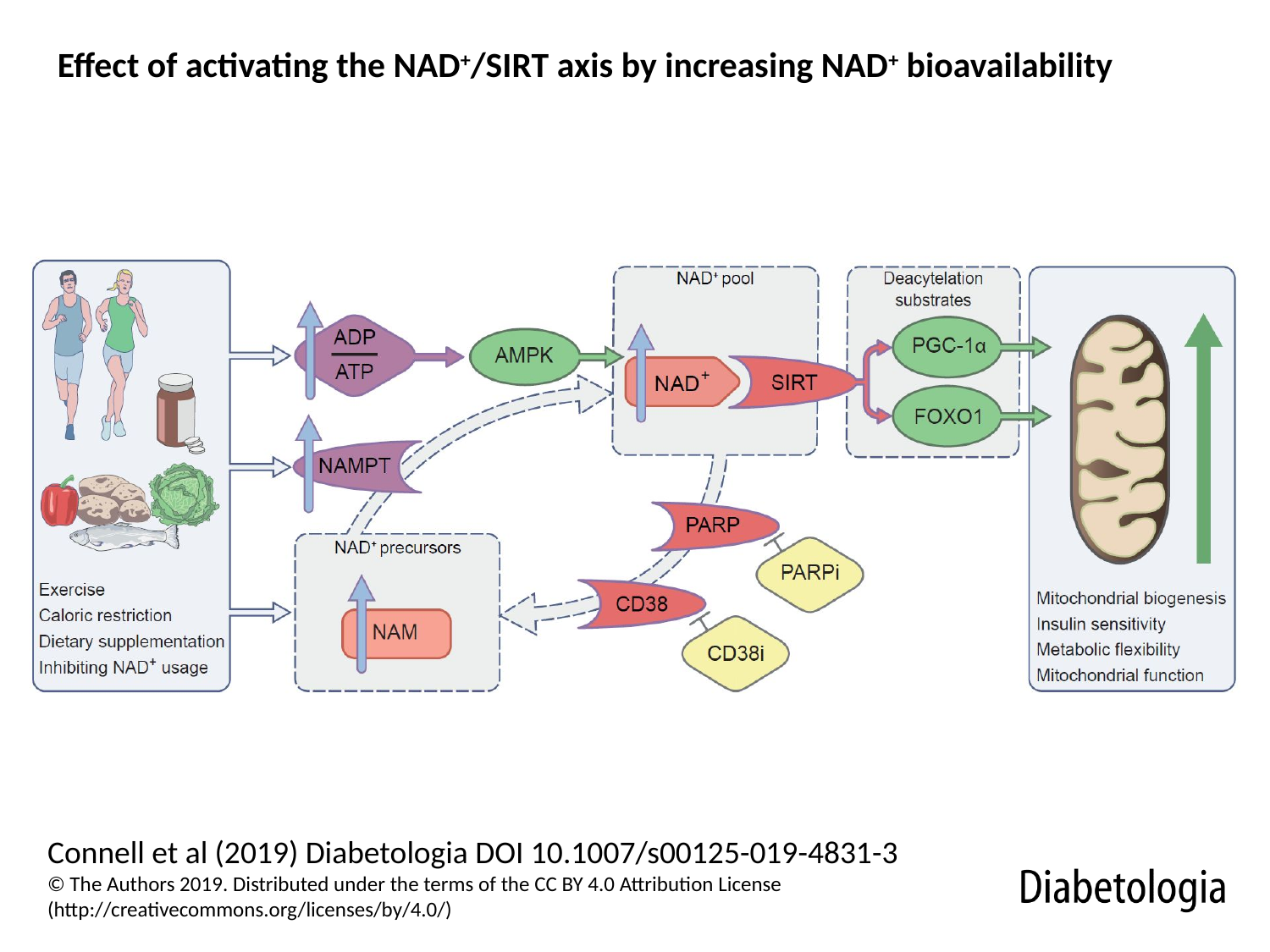

Effect of activating the NAD+/SIRT axis by increasing NAD+ bioavailability
Connell et al (2019) Diabetologia DOI 10.1007/s00125-019-4831-3
© The Authors 2019. Distributed under the terms of the CC BY 4.0 Attribution License (http://creativecommons.org/licenses/by/4.0/)
